# Supplementary material for: Are carbon emissions trading and green financial instruments synergistic? -Comprehensive quantitative research based on content analysis
Source: PLoS One. 2024 Mar 7;19(3):e0298601. doi: 10.1371/journal.pone.0298601 (PMC10919601; doi:10.1371/journal.pone.0298601)
Supplement: S1 Data — (ZIP) [file pone.0298601.s001.zip › Data/地级市Z_F工作报告.zip!/╜¡╬≈/╨┬╙α╩╨/└┤╫╘╣1⁄2╓┌║┼í╛┬φ┐╦╩2╛▌═°í┐_2015.docx]

各位代表：

　　现在，我代表市人民政府向大会报告政府工作，请予审议，并请各位市政协委员和列席会议的同志提出意见。

　　一、2014年工作回顾

　　2014年，在省委、省政府和市委的正确领导下，全市上下牢牢把握稳中求进工作总基调，坚持发展至上、富民为先，积极应对宏观经济下行压力，困难面前不退缩，矛盾面前不回避，奋力迈出发展升级新步伐，改革创新亮点纷呈，经济运行稳中向好，社会事业全面进步，人民群众幸福感和满意度不断提升。全年实现生产总值900.3亿元，增长8.8%；财政总收入126.4亿元，增长5.4%，其中公共财政预算收入89.9亿元，增长6.4%；规模以上工业增加值331.5亿元，增长11.3%，主营业务收入1670.5亿元，增长7.3%；固定资产投资748.2亿元，增长6.3%；社会消费品零售总额191.1亿元，增长11.9%；出口总额12.6亿美元，增长17.9%；实际利用外资3.5亿美元，增长10%，引进内资620.9亿元，增长16.7%；城镇居民人均可支配收入27626元，增长10.4%，农村居民人均可支配收入12831元，增长11%；居民消费价格涨幅控制在1.4%。

　　㈠凝心聚力促改革，体制机制焕发新活力。坚持以改革促发展，39项改革基本完成，18项改革稳步推进。全面推进“小政府”改革。加快转变政府职能，承接国务院、省政府取消和下放的行政审批事项252项；政府工作部门机构改革基本完成，“三区合一”改革全部到位，撤销驻外机构4个。公务用车改革成效显著，全市节约直接行政成本3059万元。在全省率先清理、规范机关事业单位编外用工，年节省人员经费725万元。推进财政金融体制改革，加强预算管理和政府性债务管控，市本级“三公”经费实际支出下降48.7%。全面推进“大社会”改革。实施了户籍管理制度改革，完成了社区管理体制改革，启动了社区数字化综合管理服务平台建设，在10个社区开展了标准化建设试点，在24个小区开展了社区管理物业化试点。市直部分中学学区制改革试点和城市公立医院综合改革初显成效，择校热、看病贵等问题得到一定缓解。全面推进“活市场”改革。充分发挥市场在资源配置中的决定性作用，推行了工商注册资本登记制度改革。在全省率先建成了企业信用和金融服务一体化平台。农村土地承包经营权确权登记颁证工作全面推进，建成了农村土地流转服务中心和管理信息平台。扩权强镇改革基本到位，计划单列镇统筹镇域经济社会发展能力进一步增强。全面推进“优环境”改革。着力创优发展环境，推行“三单”管理改革，完成了清单的收集梳理、复核审查、平台录入、网上链接、权力清单流程再造和程序固化。完成了中介机构的清理整顿并建成了网上“中介超市”，入驻中介机构137家。实行重点项目审批代办制；优化建设工程项目网上并联审批系统，共办理项目350个，行政效能进一步提升。

　　㈡坚定不移调结构，转型升级迈出新步伐。工业经济提质发展。坚持从扩大产业基金规模、加大企业帮扶力度、加快工业地产建设入手，推动工业经济调结构稳增长。新钢实现连续盈利，赛维生产经营持续向好。恩达麻世纪、沃格光电完成上市辅导备案，8家中小微企业与主办券商签约。建成标准厂房103万平方米，入驻企业76家，投产46家。新增入规企业54户。出台促进光电信息产业加快发展意见，已集聚光电信息企业70家，实现主营业务收入超30亿元。竣工光伏并网发电项目容量81.5兆瓦。分别组建了钢铁、新能源、新材料三大产业联盟，有力地推动了联盟成员间在业务、技术、项目等方面的协作，形成了三大产业集群发展新格局。第三产业加快发展。坚持以优化布局、提升水平、扩大总量为着力点，不断提升服务业的支撑带动作用。第三产业实现增加值325.4亿元，增长5.6%，占生产总值36.1%，提高0.2个百分点。各项融资余额1323.1亿元，增长10.3%，其中贷款余额909亿元，增长13.6%。发放财园通、农贷通、科贷通贷款8亿元，市国信担保公司注册资本金增至5亿元。成功发行公司债券24亿元。新注册电商企业75家，全行业实现交易额103亿元，增长69.4%。新增物流企业48家，陆路口岸作业区项目开工。夏布绣列入国家级非物质文化遗产扩展名录，恩达麻世纪列为省级文化产业示范基地。成功举办中国（江西）国际麻纺博览会、全省首届“天工杯”工业设计大赛、第三届傅抱石文化艺术节，有力促进了麻纺产业、工业设计和文化产业发展。积极策应旅游强省战略，出台促进旅游产业发展意见，启动仙女湖创建国家5A级景区工作，成功创评五星级旅游饭店1家，全市接待游客、实现旅游总收入分别增长23.8%和28%。科技创新助推发展。坚持创新驱动战略不动摇，加快推进科技创新体系建设。国家光伏工程技术研究中心、省船用钢工程技术研究中心、省民爆器材工程技术研究中心通过评估验收；国家光伏高新技术产业化基地、国家锂材料及应用高新技术产业化基地、国家螺杆膨胀动力机高新技术产业化基地通过复核；申报省级研发平台8个，组建市级研发平台5个。获批国家级高新技术企业9家。争取国家级科技项目12项、省级55项，开发省级重点新产品39项。专利申请量1194件、授权量727件，分别增长77.2%和96%。1人获国务院特殊津贴，2人获省政府特殊津贴。

　　㈢全力以赴扩投入，发展动力增添新引擎。招商引资成效明显。坚持创新招商方式、拓宽引资渠道、优化考核办法，千方百计提高招商引资工作实效。签约项目177个、金额206.6亿元，其中亿元以上项目51个、金额144.6亿元。开工项目128个，开工率79.5%；投产项目60个，投产率37.3%。重点项目进展顺利。坚持分级管理，强化日常调度，全力推进重点项目建设。57个重点项目完成投资50.7亿元。新余北站投入使用，杭南长客运专线新余段通车运营。开元制药、生辉环保建筑废弃物利用、维骏科技触摸屏等项目投产运行。政策资金争取得力。坚持开拓思路跑项目、创新方式争资金，全年共争取上级资金43.7亿元。“三项政策”累计获得上级补助资金23.7亿元。我市被列为信息惠民国家试点城市、国家信息消费试点市，高新区列入全国第一批低碳工业园区试点。煤储基地项目纳入蒙华铁路正线同步获批，大唐新余电厂2台660兆瓦机组、中电投分宜电厂2台660兆瓦机组项目获国家批准。孔目江流域综合治理项目列入亚行贷款项目规划。

　　㈣齐心协力促统筹，城乡建设呈现新面貌。规划体系不断健全。坚持规划先行，加快构建科学合理、系统完备的城乡建设规划体系。编制完成区域性详规12个，乡镇总规2个、控规12个，“五山”保护利用、中心城区基础教育设施布点等专项规划13个，村庄规划编制率达81.5%。基础设施不断完善。坚持量力而行、务求实效，不断提升城市承载能力。樟排线东段二期、滨江路、钟灵大道全面通车，滨江路西延、礼祥里路垫层通车，阳光大道西延段完成主体工程，纵三路下穿工程、公园北路建设加快推进；完成国、省道大中修64.7公里，维修县、乡道69公里，新建农村公路175公里。优先发展公共交通，新增和优化公交线路25条，增加公交里程144.2公里，新建公交站台32个，新增新能源公交车40辆。主城区新增停车泊位1514个，新建天然气加气站2个。新建和改造城区农贸市场6个。廖家江改道工程稳步推进。新建雨污管网6公里。农业农村经济不断发展。坚持大力发展现代农业，加快培育新型农业经营主体，不断提高农业经济规模化、集约化水平。粮食生产实现“十一连丰”，新余蜜桔产销两旺。新增国家级农民专业合作示范社4家、省级农业产业化龙头企业5家、家庭农场505家。省级以上龙头企业实现销售收入121.6亿元，带动20.5万农户增收5.9亿元。造林6.4万亩，林下经济面积46万亩，林地流转20.5万亩。生猪养殖生态化改造扎实推进。病险水库除险加固、小农水重点县项目年度建设任务完成。建设高标准农田3万亩。19个中心示范村建设初见成效。实施农村安全饮水项目9个，惠及2万人。罗坊沼气集中供气工程一期、下村管道天然气工程主体完工，惠及1.7万户。生态环境不断改善。坚持加大项目减排和环境综合治理力度，切实提高生态文明建设水平。提前一年完成省政府下达的“十二五”减排任务，二氧化硫、化学需氧量、氨氮、氮氧化物分别削减6.4%、1.2%、6.05%和4.59%，减排工作列全省第一。新钢4号烧结机和分电8号、9号机组烟气脱硫，新电2号机组、海螺2号生产线烟气脱硝等改造工程建成投运。完成了环境监测第三方运营等4个合同环境服务试点项目。淘汰黄标车及老旧机动车50200辆。在全省率先开展城区4吨以下燃煤锅炉整治。比国家规定提前1年监测并发布空气质量指数，全市空气质量优良率达89%；城市饮用水水质达标率100%。城区新增绿地面积4.3万平方米。“五山”保护成效初显。新建成国家级生态乡镇8个，省级生态乡镇4个、生态村6个。

　　㈤全心全意惠民生，社会事业取得新进步。坚持把保障和改善民生摆在突出位置，60件民生实事全面完成。社会保障更加有力。在全省率先发放城镇居民社会保障卡，率先推行“多险合一”信息系统试点，73.6%的参保人员实现各项社会保险“同人同城同库”一卡管理。企业退休人员养老金待遇“十连提”。新农合参保筹资标准提高到每人每年390元。发放低保资金1.6亿元，实现城乡低保应保尽保。发放临时救助资金302万元，医疗救助资金4670万元，其中重特大疾病医疗救助金417万元，惠及困难群众14464人次。开工新建廉租住房1800套、公共租赁住房5028套，发放租赁补贴6684户、1126.7万元，改造棚户区7979户、农村危房1660户。就业创业成效明显。新增城镇就业2.2万人，新增转移农村劳动力1.6万人，零就业家庭就业安置率100%，城镇就业率稳定在96.7%。新发放小额贷款4.7亿元，扶持和带动就业创业1.57万人次。社会事业蓬勃发展。学前三年毛入园率、义务教育阶段巩固率、高中阶段毛入学率分别高于全国水平16.5、2.4和2.5个百分点。在全省率先完成中小学教师职称改革试点。青少年科技创新和少儿艺术在全国赛事上获多项大奖。国家公共文化服务体系示范区创建工作进展顺利。人口健康信息综合管理平台、市人民医院儿科综合大楼建设全面启动，村卫生室标准化建设稳步推进；市直公立医院药品实现统一采购管理。“单独两孩”政策全面落实。食品药品信息化监控平台建成，群众饮食用药安全监管力度加大。在省第十四届运动会上获金牌总数列全省第四。社会治理不断加强。全国文明城市创建工作有序推进，城市管理效能稳步提高。志愿服务工作蓬勃开展，全市实名注册志愿者7.6万人。法治新余和平安新余建设深入推进，“智慧天网工程”建设成效明显，国家安全人民防线、信访维稳进一步加强，没有发生重大及以上安全事故，没有发生影响稳定的群体性事件，没有发生影响恶劣的重特大刑事案件，全市社会和谐稳定。统计、审计、海关、检验检疫、民族宗教、外事侨务、涉台事务、档案、地方志、机关事务、防灾减灾、扶贫开发、妇女儿童、青少年、老龄、科普、气象、人防、邮政通讯、烟草、盐业、海事、国防动员、民兵预备役、双拥优抚、社科研究、红十字会人道援助和慈善、残疾人事业等工作取得新成绩。

　　各位代表，2014年，我们坚持依法行政，进一步完善了政府议事规则、行政决策、责任追究等方面的工作机制，有力地推动了行政权力在法治轨道上运行。我们坚持问题导向，深入开展党的群众路线教育实践活动，建立完善了党员干部直接联系群众、党政机关公务接待管理等18项制度，开展了文山会海、民生资金使用管理等12项专项整治，集中解决了一批人民群众反映强烈的热点难点问题，形成了反“四风”、改作风的新常态。我们坚持反腐倡廉，着力强化土地和矿产资源开发、国有资产转让、建设工程招投标等方面的管理和监督，坚决查办了一批违纪违法案件，政风行风有了明显好转，政府自身建设不断加强。

　　在宏观经济持续低迷，改革发展困难重重的情况下，能够取得这样的成绩，实属不易。这得益于中央、省委省政府和市委的坚强领导，得益于市人大的法律监督和市政协的民主监督，得益于社会各界和全市广大人民群众的积极作为。在此，我代表市人民政府，向全市广大工人、农民、知识分子、干部和历任老领导、老同志，向各民主党派、工商联、无党派和社会各界人士，向驻市人民解放军、武警官兵和公安干警，向驻市中央和省属单位，致以崇高的敬意！向所有关心、支持新余发展的同志们、朋友们，表示衷心的感谢！

　　回顾过去一年的工作，我们清醒地看到，全市经济社会发展仍面临不少困难和问题：工业主导产业脱困压力重重，战略性新兴产业规模不大，产业集聚度不高，企业信用风险加大；支撑性、引领性的好项目、大项目不多，经济发展后劲还不足；城市功能有待提升，教育、医疗、养老等公共服务水平与群众需求还有不小差距，生态环境改善还有很多工作要做；财税增长依旧乏力，政府性债务潜在风险较大；社会转型期各类矛盾日益显现，各种历史遗留问题错综复杂；一些干部“为官不为”、责任意识不强、法治能力不足等问题还不同程度存在。对此，我们一定认真对待，切实加以解决。

　　二、2015年工作安排

　　2015年，是全面深化改革的关键之年，是全面推进依法治国的开局之年，也是全面完成“十二五”规划的收官之年。政府工作的总体要求是：全面贯彻落实党的十八届四中全会、中央和全省经济工作会议、市委七届十六次全会精神，坚持稳中求进工作总基调，贯彻“发展至上、富民为先”总思路，坚持以提高经济发展质量和效益为中心，以改革创新为统领，以依法行政为准绳，主动适应经济发展新常态，把握发展升级主旋律，着力抓好改革、发展、稳定各项工作，为提前全面建成小康社会打下坚实基础。

　　全市经济社会发展的主要预期目标是：生产总值增长8.5%，财政总收入、公共财政预算收入均增长5%，规模以上工业增加值增长10.6%，固定资产投资增长10%，社会消费品零售总额增长11.5%，外贸出口总额增长5%，实际利用外资增长4%、引进内资增长13%，城镇居民和农村居民人均可支配收入分别增长10%和11%。居民消费价格指数、人口自然增长率控制在省定目标以内，完成省下达的节能减排计划任务。

　　围绕上述目标，重点做好以下工作：

　　㈠坚持改革创新不动摇，着力激发社会发展活力

　　深化行政管理体制改革。加快推进政府职能转变，实行行政审批事项目录管理制度，积极对接落实国务院和省政府下放、取消的审批事项，推行行政审批和社会服务事项网上办理，完成县区“三单”管理改革。建立重大支出依法申报、集体决策、按期报告制度，将非税收入纳入预算管理。全面推进预算绩效目标管理，逐步实现财政支出绩效评价全覆盖。全面清理、整合、规范使用市级财政专项资金。

　　深化市场体制机制改革。继续推进投融资管理改革，全面清理、规范投融资平台。强化政府性债务“融、用、管、还”管理，建立偿债准备基金，控制债务规模，防控债务风险。整合土地和矿业权交易、建设工程招投标、政府采购、产权交易四个要素市场，升级建设覆盖市、县、乡三级的公共资源网上交易平台。出台政府购买服务管理办法和指导目录，逐步建立政府购买服务新机制。

　　深化社会治理体制改革。出台社会信用体系建设规划纲要，以企业和公务人员为重点，逐步扩大征信范围，加快建设覆盖全社会的信用体系。推进企业信用与金融服务一体化平台运用，健全守信激励和失信惩戒联动机制。推动志愿服务工作向社区、乡村等基层延伸，不断完善志愿服务工作体系，实现志愿服务工作科学化、规范化、常态化发展。积极预防和化解各类社会矛盾，推进涉法涉诉信访工作改革，实行诉访分离。

　　深化民生事业改革。加快推进社区标准化建设，完善网上便民服务系统，在全省率先建成社区数字化综合管理服务平台，将公众服务事项网上办理延伸到社区，为居民提供方便快捷的一网式、多渠道服务，实现社区综合管理的扁平化、网格化和智能化。全面推行村级民事代办制。深入推进教育领域综合改革、城市公立医院综合改革。

　　㈡坚持工业主导不动摇，全力夯实经济发展基础

　　推进工业转型升级。围绕抓龙头、补链条、创名牌、拓市场，培育壮大钢铁及机械装备制造、光伏、新材料、光电信息等产业集群，努力形成产业发展新优势。充分发挥钢铁产品优势，大力发展机械装备制造业。着力推动光伏产业向电站设计、电站安装、电站建设等领域延伸，提高产业集聚度。加快引进和培育光电信息等新兴产业，力争实现光电信息企业超100家、主营业务收入超100亿元。继续举全市之力支持园区建设，落实好支持园区发展的各项政策，深化园区企业化管理改革，加强园区管网、保障性住房等基础和配套设施建设，鼓励社会资本投资建设工业地产，不断提升园区承载能力。力争全市新增入规企业50户。

　　加大企业帮扶力度。继续实行领导挂点帮扶企业制度，对部分重点企业派驻帮扶工作组，“一企一策”化解企业在信用风险、经营管理、法律纠纷、融资用工等方面存在的困难和问题。设立还贷周转金，整合政府性担保机构，建立担保风险代偿和补贴机制，充分发挥财园通、农贷通、科贷通作用，着力防范化解企业信用风险。深化校企对接合作，有效缓解企业用工难题。完善产业联盟运行机制，鼓励建立产业技术创新战略联盟，提高企业可持续发展能力。全力支持新钢改革试点。

　　提升创新驱动能力。深入实施科技协同创新“六大引领工程”，加大国家创新型试点城市创建力度。引导企业加大研发投入，提高科技成果产业化水平。力争新增高新技术企业5家以上，获批国家级和省级科技项目分别达5个、40个以上，推广新技术、新品种项目50项。深化与高等院校、科研机构合作，力争孵化科技成果超20项。推进专利开发转化，力争实现专利申请量1200件、授权量600件。加大人才政策和机制创新力度，重点引进科技创新人才、企业管理人才和高层次人才。

　　㈢坚持投资拉动不动摇，强力推进重点项目建设

　　推进重点项目建设。计划安排1000万元以上投资项目102个，总投资280亿元，年度计划投资49.3亿元，建成投产项目48个。继续坚持重点项目领导挂点制、项目建设分级管理制、政府投资项目代建制，加快推进重点项目投产投运。重点项目分批次下达，首批计划安排40个，年度计划投资33.8亿元，其中新建项目26个，年度计划投资21.8亿元。全力配合蒙华铁路新余段建设，争取煤储基地项目年底前开工，完成72兆瓦分布式光伏发电示范区建设，举全市之力推进大唐新余电厂、中电投分宜电厂两个火电项目年内开工。深入推进“三项政策”项目的争取和实施；积极推进与央企对接合作；重点围绕国家及省“7+2”重大工程项目计划，加强项目编制，加大向上争资金、争项目、争政策力度，力争争取上级资金增长10%以上。

　　提高招商引资实效。突出领导引领招商，落实“一把手”招商责任制，力争签约引进项目150个以上。强化主导产业招商，注重招大引强，紧盯光电信息、机械装备制造、文化旅游等重点领域，围绕上下游配套，实行定向、定点、定单专题招商。积极探索商（协）会招商、以商招商和委托招商，大力推进新余籍人士回乡创业、本土创业。健全招商项目跟踪落实和考核奖惩机制，促进项目早落地、早开工、早投产。精炼专业招商，全市成立30支专业招商队伍、开辟30个委托招商点、聘请30名委托招商人，切实提升专业招商队伍的素质和水平。

　　有效释放社会投资。坚持市场化运作，充分发挥政府性投资的引导带动作用，真正让社会资本成为投资主体。政府投资主要投向公益性和基础性项目建设，对竞争性领域的产业，政府由直接投资改为设立投资基金，吸引社会资本投入。大力推行政府与社会资本合作（PPP）等多种投资模式，创新投融资机制。充分发挥财政资金的杠杆作用，吸引民间资本参与兴办教育、医疗、养老等公共事业和环境保护等基础设施建设，加快建立多元化可持续的资金供给体系。

　　㈣坚持调优结构不动摇，努力做大做强第三产业

　　重点发展生产性服务业。加快金融机构引进，力争新设村镇银行1家。拓宽融资渠道，力争1家以上企业在“新三板”挂牌，推动担保机构和小额贷款公司增资扩股。创优融资环境，严厉打击恶意逃废债务和非法揽储行为，严格落实各项信贷优惠政策。加强政银企协作，优化信贷结构，确保年末各项融资余额1400亿元，其中各项贷款余额970亿元，均增长8%以上。加快推进物流综合信息平台建设；以赣西中心物流园区、高新区物流中心建设为依托，着力培育龙头物流企业。促进工业设计与实体经济融合发展，提高工业设计产业化水平。

　　提升发展消费性服务业。积极推进社区商业网点建设，新建、改造城区农贸市场3家，新增净菜超市30家、放心粮油配送网点5家。引导餐饮企业创新经营方式，着力培育有品位、有特色的小吃项目。扎实推进农村电子商务建设，完善农村商贸流通体系。完成旅游总体规划修编，加大旅游项目策划包装、引进建设力度，深度开发罗坊红色旅游资源，大力发展运动体验、养生康体、乡村休闲等旅游新业态。推进仙女湖国家5A级景区和昌坊4A级景区创建，力促亚热带植物园、中国洞都等新景区景点开业。强化旅游宣传推介，办好爱情文化旅游节等主题活动。实现接待游客增长20%、旅游收入增长25%以上。

　　创新发展新兴服务业。加快推进金利达、全城电商等电商基地建设，力争新增电商企业20家以上。大力发展文化创意、传媒出版、文化产品制造等文化产业，力争增加值突破30亿元。积极推进抱石文化创意园、恩达夏布文化创意馆等文化园区建设，提升文化产业承载能力。重点支持动漫、夏布绣等文化企业发展，加快培育一批具有自主知识产权的优势企业。继续办好麻纺博览会，推动苎麻产业加快发展。加快发展法律、会计审计、咨询评估等中介服务业，大力发展养老托幼、家政服务、家庭教育等服务业。

　　㈤坚持城乡统筹不动摇，大力推动城乡一体发展

　　建设“精美特新”中小城市。全面启动全国文明城市创建工作，提升城市品味，建设美丽新余。理顺城乡规划管理体制，实现城乡规划管理一体化。完善城乡规划编制体系，启动城市总规实施评估和修编工作，抓好北湖、新钢、城南三个片区详规和消防等专项规划编制。围绕解决行路难、停车难问题，改造公园路，打通五金路，完成长青北路标准化改造，实施新欣大道和天工大道路面维修，拓宽3个交叉路口，建成货运车辆进入主城区24小时监控系统，完善交通信号灯和标志标线设置，新增城区公共停车泊位1500个。围绕解决群众居住环境不优、生活不便的问题，注重城市细节完善和管理，推进10个老旧小区、10条背街小巷标准化改造，加大老旧供水、供气管网改造力度，实施配电网“手拉手”工程，完成20个小区物业管理标准化建设。围绕解决城市内涝问题，完成廖家江改道一期工程建设和贯早江排水渠改造，启动廖家江改道二期工程建设，抓好30公里排水管网缺陷修复。推进袁河生态新城核心区江心洲片区建设，完善高铁新区基础设施。合理引导刚性需求，推动房地产市场平稳健康发展。加大土地收储力度，注重城市土地有序供应，切实提高供地率和土地利用率。建立完善城市管理协调联动机制，推进数字城管信息采集和坐席受理等服务外包，提升城市管理精细化、规范化、智能化水平。

　　推进镇村联动建设。完善城乡路网体系，加快欧里至东岳庙公路及人和至洞村连接线工程建设，启动欧里至双林公路改造，完成上吉线、石镇线、樟排线等养护大中修工程；加大农村危桥改造力度，新建农村水泥公路100公里。启动高铁新区长途客运站和仙来公交枢纽站建设，开通城区至观巢、欧里等公交线路，推进城乡客运一体化。切实抓好计划单列镇和19个中心示范村、190个新农村建设点建设，积极推进古村落、古建筑保护利用工作。加大农村环境整治力度，突出抓好垃圾处理和污水治理，建立和完善市、县（区）、乡（镇）、村四级城乡生活垃圾收运处理体系。抓好农村安全饮水项目实施，解决农村4.3万人饮水安全问题。

　　加快发展现代农业。以农民增收为核心，加大农业科技创新力度，大力发展休闲观光农业和生态循环农业，推进农业结构调整，提高农业综合效益。培育发展加工型农业产业化龙头企业，提高农产品附加值和品牌竞争力。力争新增年销售收入超10亿元的农业产业化龙头企业1家、超亿元的2家。大力培育种养大户、家庭农场等新型经营主体。基本完成农村土地承包经营权确权颁证工作，有序推进农村土地承包经营权流转，发展适度规模经营。抓好现代农业示范园核心区和“一村一品”示范点建设。积极稳妥推进林地流转，大力发展林下经济。抓好37座病险水库除险加固、小农水重点县、中小河流治理及水系连通等项目建设，治理水土流失面积3.6万亩，建设高标准农田3万亩。

　　㈥坚持环境保护不动摇，致力提升生态文明水平

　　加强环境综合治理。开展工业废气、燃煤烟气、机动车尾气、餐饮油烟和道路扬尘污染专项整治，实施城区10吨以下工业燃煤锅炉整治，努力改善大气环境质量。抓好全国水生态文明城市建设试点工作，实施仙女湖、孔目江集中饮用水源地水质保护和24小时监测，确保水质稳定在Ⅱ类标准。继续推进“森林城乡、绿色通道”建设，完成造林绿化5万亩、森林抚育6万亩。做好“五山”保护利用和生态修复工作。

　　扎实推进节能减排。抓好钢铁、电力、水泥等行业重点企业脱硫、脱硝、除尘设施运行管理，加快新电1号机组、分电8号和9号机组脱硝工程项目建设。严格实施建设项目环境影响评价和环保“三同时”制度，加大重点行业清洁生产技术改造力度。加快淘汰黄标车、老旧机动车，积极推广使用液化天然气、混合动力等清洁能源汽车，新增节能减排公交车20辆，着力构建城市绿色交通体系。深入推进合同环境服务试点，抓好城西污水处理厂和生活垃圾焚烧发电项目建设。

　　完善生态文明建设机制。全面落实全省生态文明先行示范区建设要求，着力构建节约资源和保护环境的空间格局、产业结构和生产生活方式。完善县区综合考核评价体系，进一步提升生态文明建设权重。开展领导干部自然资源资产离任审计试点。完善森林生态效益补偿基金制度，健全矿产资源有偿使用、矿山环境综合治理和生态恢复保证金制度。

　　㈦坚持富民为先不动摇，倾力发展各项社会事业

　　保障改善民生。按照“保基本、兜底线、促公平”的原则，不断加大民生投入力度，全力办好30件民生实事，让人民群众共享改革发展成果。坚持把促进就业创业放在经济社会发展优先位置，新增城镇就业人员2.16万人，就业率稳定在94.5%。扩大小额贷款扶持范围，新增再就业小额贷款3.8亿元。积极推进“市民基础保障包”建设，扩大社会保险覆盖面。完成市养老服务中心一期工程建设。提高企业退休人员基本养老金和城乡居民基础养老金水平，实施机关事业单位养老保险制度改革，启动城乡居民基本医疗保险制度整合工作。全面推行“助保贷款”政策，帮助困难人员接续养老保险关系。实现城乡低保应保尽保，实施无支付能力患者疾病应急救助，切实提高社会救助工作水平。以产业扶贫为抓手，扎实推进35个贫困村扶贫攻坚。推进保障房由中心城区向工业园区布点，落实县区建设主体责任。新建公租房1485套，发放租赁补贴6000户，改造中心城区棚户区3637户、农村危房1300户。

　　发展社会事业。完成城区5所小学扩容改造，新增学位7200个。进一步扩大优质教育资源覆盖面，不断缩小城乡、校际间办学差距。支持新余学院转型发展；大力发展职业教育，深化现代学徒制试点，开展校企合作、集团化办学，提升服务地方经济发展能力。全面完成国家公共文化服务体系示范区创建任务，实现公共文化服务“全省领先、中部示范”目标。实现图书馆等五大公益性场馆向社会免费开放。加快卫生重点学科建设与高层次人才引进培养，加快医疗新技术、新项目开发与应用。完善突发公共卫生事件应急指挥平台，完成人口健康信息管理技术支撑平台建设。切实保障群众饮食用药安全。加大体育设施建设投入，组织开展100项全民健身活动。充分发挥各方面智慧，科学编制“十三五”规划。切实抓好精神文明建设。关心支持妇儿、老龄事业，发展残疾人事业和慈善事业。加强国防后备力量和人民防空建设，推进“双拥”和优抚安置，巩固和发展军政军民团结。做好新形势下的科普、气象、史志、档案、外事侨务、民族宗教、驻外机构、防震减灾、邮政通讯等工作。

　　建设平安新余。大力推进依法治市工作，加强律师队伍建设，提高法律工作者服务水平。加强国家安全人民防线建设和应急管理工作。积极构建城乡治安防控体系，新建改建城区治安岗亭9个，提高街面见警率，防范和打击各类违法犯罪，全力维护社会政治稳定，提高人民群众安全感。强化安全生产责任落实，深化重点行业领域隐患排查治理和安全生产专项整治，预防重特大安全事故发生。

　　各位代表！我市改革、发展、稳定的任务十分艰巨和繁重，对政府工作提出了更高的要求。我们将坚持“为民、务实、高效、廉洁”原则，切实加强自身建设，不断提高政府的执行力和公信力，全心全意为人民服务、对人民负责、让人民满意。全力打造法治政府。坚持在法治轨道上开展工作，运用法治思维和法治方式深化改革、推动发展。坚持“法定职责必须为，法无授权不可为”，科学厘清各级政府及其部门的职能与权限，坚决纠正行政不作为、乱作为，坚决惩处失职、渎职。坚持以公开为常态、不公开为例外原则，推进决策公开、执行公开、管理公开、服务公开、结果公开，重点推进财政预决算、公共资源配置、重大建设项目批准和实施、社会公益事业建设等领域的政府信息公开。健全依法决策机制，进一步规范重大决策法定程序，健全重大决策合法性审查制度，完善重大决策风险评估机制，实行重大决策终身责任追究制度及责任倒查机制。加快建立政府法律顾问队伍。深化行政执法体制改革，清理和整合行政执法队伍，杜绝多层、多头执法。严格规范执法行为，全面落实行政裁量权基准制度和行政执法责任制度。自觉接受人大、政协监督，强化监察、审计监督，高度重视社会舆论监督。持续推进作风建设。始终绷紧反“四风”这根弦，在思想上不松懈，在行动上不松劲，不断巩固和扩大群众路线教育实践活动成果。坚决维护制度的权威性，做到令行禁止、不搞例外，以制度的刚性约束根治作风之弊、行为之垢。认真落实首问负责制、限时办结制、服务承诺制等制度，切实提高工作效能。坚持从群众中来，到群众中去，深入开展调查研究，掌握实情、解决问题、推动工作。严格履行向社会作出的承诺，以实实在在的工作成效发展好、维护好、实现好人民群众的根本利益。始终保持清正廉洁。大力弘扬勤俭办事业的优良传统，牢固树立过紧日子思想，珍惜民资民力，严禁铺张浪费，坚决压减行政成本，市本级一般性支出预算再压减10%，集中财力促发展、办大事、保民生。守纪律、讲规矩，切实履行党风廉政建设主体责任，严格惩治和预防腐败。深入开展损害群众利益突出问题专项整治，坚决纠正行业和部门不正之风。严格执行土地出让、工程招标、政府采购等各项规定，以风清气正的廉洁形象，赢得人民群众的信赖和支持。

　　各位代表，新的一年面临新的挑战，也将迎来新的机遇。让我们紧密团结在以习近平同志为总书记的党中央周围，在省委、省政府和市委的坚强领导下，敢于担当，锐意进取，齐心协力，攻坚克难，为加快建设和谐富裕文明新余、提前全面建成小康社会而努力奋斗！
